# From Pregnancy to Postpartum: The Cardiovascular Risks Associated with Gestational Diabetes

**DOI:** 10.2174/011573403X354645250329171327

**Published:** 2025-04-17

**Authors:** Ramsha Sharma, Ujjawal Singh, Raj Kamal, Ranjeet Kumar

**Affiliations:** 1 Department of Pharmacy Practice, ISF College of Pharmacy, Moga, India;; 2 Department of Pharmaceutical Quality Assurance, ISF College of Pharmacy, Moga, India;; 3 Narayan Institute of Pharmacy, Gopal Narayan Singh University, Sasaram, Rohtas, Bihar, 821305, India

**Keywords:** Cardiovascular disease, postpartum cardiovascular disease, gestational diabetes mellitus, maternal health, gestational hyperandrogenism, pregnancy

## Abstract

Cardiovascular disease is the leading cause of pregnancy-related mortality, with pregnancy-related cardiovascular issues extending into the postpartum period. Recent studies suggest hyperandrogenism alters sex hormone levels, contributing to gestational cardiovascular disease CVD. Most of the factors behind the onset of CVD in postpartum women remain unknown. Animal studies mimic adverse pregnancy outcomes to explore molecular causes of severe prenatal cardiac events and their role in postpartum cardiovascular disease development. This review will be focused on summarising human and animal research that shows how undesirable pregnancy outcomes, such as obesity in the mother and gestational diabetes (GD), have an impact on postpartum cardiovascular disease and prenatal cardiometabolic dysfunction. We will highlight the adverse effects of gestational hyperandrogenism as a potential biomarker for cardiovascular dysfunction in pregnant women and new mothers. Investigative cardiovascular (CV) risk variables in the early postpartum phase following pregnancy that were impacted by GD was the aim of this study. Current research strongly implies that women with GDM have a higher risk of developing CVD. Finding appropriate, reliable indicators of CVD and specific treatment modalities that can control obesity, diabetes, and metabolic syndrome are critical to reducing the burden of CVD on impacted women. GD and hypertensive disorders are two pregnancy-related illnesses that raise the risk of CVD in the long run. Despite a lack of awareness, early screening, lifelong monitoring, and continuous research to enhance detection and prevention are essential.

## 1 INTRODUCTION

The diagnosis of gestational diabetes mellitus (GDM), which is characterized by the development of glucose intolerance during pregnancy, has been the subject of debate since it was first documented in 1964 [1]. There is widespread agreement that GDM indicates a future risk of type 2 diabetes (T2D), even though ongoing debate in the intervening half-century has focused on diagnostic criteria and best screening and detection procedures. Even though a woman's glucose tolerance generally goes back to normal soon after delivery, she has a 20% to 70% chance of developing T2D over the ten years of receiving a diagnosis of GDM. Given that these women have a greater overall incidence of T2D compared to others, GDM serves as a known clinical predictor of potential diabetes risk [2]. Unlike the more than 50-year-old link between prenatal glucose and T2D, the link between cardiovascular disease (CVD) and diabetes has just recently been known. Notably, several studies conducted recently have revealed that despite being relatively young (*i.e.*, childbearing age), women with GDM have an elevated incidence of significant cardiovascular events in years to decades postpartum [3]. The risk level associated with this condition varies according to different studies, and it’s unclear whether its occurrence depends on the simultaneous development of T2D. These conflicting findings significantly influence cardiovascular risk evaluation in women. Specifically, clinical monitoring of glucose tolerance should be sufficient to induce vascular examination in women with a history of GDM if an elevated cardiovascular risk only materializes upon progression to T2D. In contrast, regardless of whether they develop T2D [4-6], all women with GDM may call for vascular surveillance if this risk is not contingent on undercurrent diabetes. Therefore, to get a trustworthy assessment of the future CVD risk in women with GDM and ascertain the role of T2D in this, we carried out a systematic review and meta-analysis for this commentary. Specifically, some studies have shown that women with GDM only experience CVD if they progress to T2D; nevertheless, other investigations have contended that an elevated risk of cardiovascular disease might appear even in the absence of diabetes [7-9]. These conflicting findings significantly impact cardiovascular risk evaluation in women. Specifically, if an increased cardiovascular risk only emerges with the onset of T2D, routine glucose tolerance monitoring should prompt vascular examination in women with a GDM history. On the other hand, all women with GDM may require attention, irrespective of whether they develop T2D. To assess the effect of type 2 diabetes (T2D) on the risk of cardiovascular disease (CVD) in the future for women with gestational diabetes mellitus (GDM), we carried out a systematic review and meta-analysis. The risk of cardiovascular disease (CVD) events, such as heart attacks, heart failure, and arterial disease, is doubled for women with a history of GDM. The risk is considerably higher for pregnant women who have higher socioeconomic health burdens.

## 2 CHRONIC HEALTH CHALLENGES AFTER GDM PREGNANCY

### 2.1 T2D Mellitus

Pregnancy-related gestational diabetes and the future onset of T2D are strongly correlated. Numerous meta-analyses have demonstrated that women who have had gestational diabetes are six to 10 times more likely to acquire T2D [10-13]. It is estimated that between 11% and 30% of women with T2D may have previously had gestational diabetes [13]. For every ten years after a diagnosis of gestational diabetes, the incidence of T2D rises by around 10%. South Asian women, in particular, seem to be at a higher risk. As a result, the prevalence of T2D during pregnancy is increasing, especially among younger women who already have T2D. In India, the rate of pregnancy complicated by pre-existing diabetes varies locally and is influenced by the ethnic makeup of the population under study. At least half of these women have T2D, which is becoming more common. GDM is typically thought to be a consequence of pancreatic beta-cell dysfunction in women who already have insulin resistance, which heightens the likelihood of T2D post-pregnancy. The identification of GDM-afflicted women who are most at risk of T2D is therefore critical from the standpoint of public health - a high-fat diet, exercise, and stress, whose incidence is fast rising globally. Aging also impairs insulin function, which in turn causes the disease to develop. The prevalence of GDM is 7% of pregnancies. There may be a substantially higher prevalence and greater propensity for diabetes in certain minority populations. Preeclampsia, uncharacteristic weight gain, and C-sections are more prevalent in patients with GDM. Children born to moms who have GDM are more likely to experience macrosomia, birth injuries, and shoulder dystocia. Postpartum respiratory distress syndrome, hyperbilirubinemia, postpartum hypoglycaemia, hypocalcaemia, polycythaemia, and later T2D and obesity are all more prevalent in these newborns [14].

### 2.2 Cardiovascular Disease

Traditional cardiovascular risk factors were more prevalent among moms with diabetes. The findings of a meta-review show a rise of triglycerides (SMD, 0.55; observed as early as a year postnatal), cholesterol in the form of low (SMD, 0.18; 94% CI, 0.08-0.20), Systolic measurements of arterial pressure (average difference, 2.44 mmHg; 94% CI, 1.71-3.34mmHg) and mass index of the body (average difference, 1.53 kg/m^2^; 95 percent CI, 1.31-2.46 kg/m^2^) [15]. When these factors are combined, the likelihood of developing Pregnancy-related diabetes in pregnant women's metabolic syndrome (MS) triples [16]. Therefore, it’s not surprising that women who have previously experienced gestational diabetes have a doubled risk of CVD events (relative risk [RR], 1.98; 95% confidence interval [CI], 1.57-2.50). This RR increased in the first decade following the diagnosis (RR, 2.31; CI, 1.57-3.39). The risk of arterial disease, heart attack, heart failure, and injury are also greater in these women (RR, 1.45; 95% CI, 1.28-1.62). This rephrased content maintains the original meaning while ensuring uniqueness [17, 18]. Currently, studies are being done to determine if prenatal metabolic malfunction of heart (CMD), which can cause postpartum CVD in women, and poor pregnancy outcomes (APOs) are related [19-25]. Preeclampsia [26-30], hypercholesterolemia [31, 32]), obesity [33], and GDM are notable examples of gestational CMD [26, 27, 31, 32], and also genetic, environmental, dietary, behavioural, and ethnic factors all play a role in the development of various CMDs [34, 35]. Similarly, women who had the greatest socioeconomic factors of health burden during pregnancy also had the highest risk of CVD as shown in Fig. (**1**) [36]. A dose-dependent relationship between parity and CVD risk was also found in a study [37].

### 2.3 Gestational Diabetes Risk Factors

**Fig. (2):**
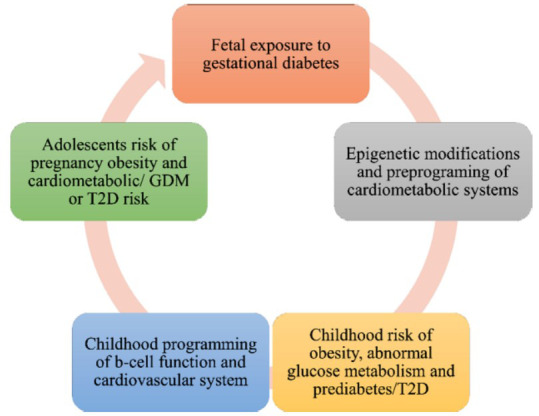
Intergenerational impact of gestational diabetes: Maternal hyperglycemia causes fetal hyperinsulinism, predisposing offspring to cardiometabolic risks like obesity, dysglycemia, dyslipidemia, hypertension, and early renal dysfunction. These issues emerge in adolescence and increase the likelihood of young women developing gestational diabetes, perpetuating the cycle.

Since postpartum testing is insufficient, screening is crucial for the early diagnosis of additional cardiovascular-related risk factors like diabetes with type 2 diabetes [38]. In conformity with worldwide conventions, the Association of Royal Australian College of Paediatricians recommends that women who have experienced gestational diabetes have a glucose tolerance test six to twelve weeks after giving birth, and then every one to three years afterwards [39, 40]. There are few risk factors for GDM, and they are frequently complicated by other conditions [41, 42]. Moreover, the task of comparing outcomes across various studies is complicated due to the differing criteria for diagnosing GDM and assessing risk factors. Nevertheless, several risk indicators for GDM persistently emerge despite these concerns. These include a Westernized diet, ethnicity, genetic variants, being overweight or obese [43], experiencing extra growth throughout pregnancy [44], being older, the intrauterine environment [45], having a family history of diabetes-related conditions, such as PCOS (polycystic ovarian syndrome) [46], and GDM [41] and being older than norm for birth weight [47]. Each of those risk factors is associated with the reduced effectiveness of β-cells and/or insulin sensitivity, whether directly or indirectly. For instance, persistent, excessive caloric intake, which overpowers insulin signalling pathways and β-cell production, is intrinsically linked to overweight and obesity (Fig. **2**).

### 2.4 Hypertension During Pregnancy

Hypertension that begins during the 20 weeks of gestation is recognized as a pregnancy-related hypertensive condition, which includes gestational hypertension and preeclampsia [27, 48]. The distinguishing factor between preeclampsia and gestational hypertension is the presence of proteinuria, which is seen in 11% to 16% of pregnancies and is linked to a significantly increased risk for CVD [26, 49]. According to recent research, established CVD risk factors such as Diabetes type 2, persistent high blood pressure, and hyperlipidaemia heighten CVD risk in pregnant women with hypertension [26]. Hypertension related to pregnancy is also associated with hyperlipidemia, persistent hypertension, along diabetes mellitus, all of which are potential CVD risk factors [27]. Preeclampsia not only increases the likelihood of hypertension post-pregnancy but also elevates the risk of CVD, encompassing peripheral vascular disease, coronary artery disease, and heart failure [23, 50, 51]. Presumptive women are four times more likely to develop cardiac failure in addition to a two-fold greater likelihood of CAD, stroke, and death [52].

### 2.5 Obesity

For females who are of reproductive age, obesity is the most prevalent medical problem [53]. On its own, pregnancy-related obesity raises the risk of maternal morbidity and mortality [54]. Because of their metabolism, obese women have a higher risk of insulin resistance throughout the first trimester of pregnancy. Eventually, this resistance develops into glucose intolerance, which increases the chance of getting GDM, a recognized risk factor for CVD throughout pregnancy and the postpartum period [33, 54, 55]. A meta-analysis of 20 cohort-based research studies has found that pregnant women of normal weight had an average two-, four, and eight-fold less chance of acquiring GDM, a recognized risk factor for heart failure, than did overweight, obese, and severely obese women [56]. Although there is little information on the precise relationship between prenatal obesity and maternal CVD, it is generally acknowledged that fetal obesity raises the risk of preeclampsia, endothelial dysfunction, GDM, gestational hypertension, and systemic inflammation, all of which are associated with an increased risk of CVD [57-59].

### 2.6 Polycystic Ovarian Syndrome

An increasing number of illnesses have recently been connected with elevated androgen production [60, 61]. As the most prevalent endocrinopathy in women of age at conception, PCOS is marked by polycystic ovaries, hyperandrogenism, and ovulatory dysfunction. Insulin resistance linked to PCOS increases polycystic ovarian resistance in pregnancy, which may worsen or develop GDM.

## 3 DIABETES IN PREGNANCY: PATHOPHYSIOLOGY

Since GDM often arises from poor cell activity in the context of long-standing resistance to insulin throughout pregnancy, the etiology of GDM involves both tissue insulin rebellion and impaired cell turnover. More often than not, these shortcomings are present before pregnancy and may advance, heightening the probability of T2D onset postpartum [62]. GDM has an impact on or contributes to a variety of different organs and systems. This group consists of the placenta, liver, muscle, adipose tissue, and the brain as shown in Fig. (**3**).

### 3.1 Dysfunction of β-cell

Response to a glucose load *via* the storage and release of insulin is the primary function of beta cells. Cell breakdown happens if there is a shortage of insulin generated by the cells reacting to a spike in the amount of glucose in the blood. It is believed that chronic fuel excess causes cells to continuously overproduce insulin, which in turn causes cell malfunction [63]. Nevertheless, specific mechanisms underlying β-cell dysfunction may vary and be intricate [64, 65]. There are several steps in the method where errors may occur, including proinsulin synthesis, posttranslational changes, granule storage, monitoring of blood glucose, and complex mechanisms controlling granule exocytosis. It comes out that glucokinase (Gck) or potassium voltage-gated channel KQT-like 1 (Kcnq1) constitutes the vast majority of genes of susceptibility linked to GDM, which alters -cell function. Microscopic defects in the cellular machinery are only visible during metabolically stressful times, like pregnancy [66]. Insulin resistance makes the functioning of β-cells worse. Because reduced insulin-stimulated glucose absorption overworks β-cells, leading them to release more insulin, it improves the possibility of hyperglycemia. The term “glucotoxicity” describes how glucose directly contributes to cell death [67]. As an outcome, once β-cell failure commences, a vicious cycle leads to β-cell failure, elevated blood sugar levels, and obesity. Thus, insufficient production of insulin by pancreatic cells in women having gestational diabetes is triggered by insulin resistance resulting from the placenta [68]. Internal granule insulin stores are depleted, and glucose-stimulated insulin production is significantly diminished in these settings due to a short-term but drastic drop in β-cell mass that kills surviving β-cells. A person's unique circumstances may lead to β-cell failure, β-cell mass decline, β-cell number damage, or a combination of all three causing GDM [69].

### 3.2 Neurohormonal Networks

Some ideas suggest that insulin-resistant diseases like GDM have a neurohormonal disruption as part of their pathophysiology. This complex web of central (brain regions controlling cognition, vision, and “reward”) and peripheral (satiety and hunger hormones) signals controls baseline metabolic rate, physical consumption of energy, and appetite [70, 71]. These variables change how body uses glucose and how fat is absorbed, which affects GDM. This system is largely governed by circadian rhythm, which could elucidate why individuals with severe sleep disorders or those working in shifts exhibit a higher prevalence of GDM. Adipokines, predominantly secreted by adipose tissue and serving as cell-signalling proteins, are key regulators of neurohormonal metabolic control. Notable among these are adiponectin and leptin [72, 73].

### 3.3 Adipose Tissue Inflammation

Conditions such as obesity, T2D, and GDM are often associated with an increased presence of adipose tissue macrophages (ATM). Pro-inflammatory cytokines, including TNF-a, IL-6, and IL-1, are known to be released by these ATMs. According to recent research, a modest inflammatory condition has been linked to the development of insulin resistance. These pro-inflammatory cytokines have been discovered to disrupt beta-cell insulin production and insulin signaling. They either cause the IRS-1 to be degraded *via* the STAT3-SOCS3 pathway, enhance serine protein phosphorylation of IRS-1, or inhibit actions of tyrosine of the insulin-related receptor (IR) [74, 75]. The levels of these pro-inflammatory cytokines in the blood are higher in GDM. There is a strong relationship between plasma TNF and difficulty metabolizing insulin [76]. But there is more to the tangled link between swelling and gestation. The relationship between low-degree chronic inflammation and GDM is not clear-cut, even though the latter condition appears to be significantly influenced by the former. This rephrased content maintains the original meaning while ensuring uniqueness.

### 3.4 Adipokines and Hormones

Adiponectin is a key player in the physiological process of sensing insulin for blood sugar. Additionally, it functions as an anti-atherogenic and anti-inflammatory to preserve immunological contact between the blood of the mother and fetus across the barrier of the placenta [77, 78]. Adiponectin may be a useful biomarker for predicting the beginning of GDM [79, 80]. Human placental lactogen (hPL), oestrogen, progesterone, cortisol, and prolactin are among substances that increase with pregnancy and may reduce peripheral insulin sensitivity, according to research [81]. This volatile metabolic condition elevates levels of blood glucose and free fatty acids, thereby promoting GDM in pregnant women. Owing to hindered IRS-1 activity and the phosphorylation process of receptors for insulin, there’s a notable increase in expression of adipocyte fatty acid binding protein, coupled with a reduction in expression of PPAP- and persistent inflammation in pregnancies associated with GDM [82].

### 3.5 Liver

The synthesis of dextrose (gluconeogenesis) in the liver is increased in GDM. In the fed condition, gluconeogenesis is insufficiently inhibited while fasting, and *vice versa* [83]. The fact that the majority of the liver’s glucose uptake (about 70%) is not insulin-dependent; it is not thought that this is completely the result of insulin resistance leading to erroneous glucose sensing. These results could be explained by shared elements, such as PI3K, between the insulin signalling system and mechanisms regulating gluconeogenesis [84]. By supplying too much gluconeogenesis substrate, increased protein consumption and muscle breakdown may also accelerate the process [85]. Despite this, T2DM or GDM doesn't seem to be primarily caused by the liver [86].

### 3.6 Oxidative Stress

An imbalance between antioxidants and antioxidant-producing cells causes oxidative stress. Oxidative stress modifies the conditions of proteins, lipids, and DNA, and it can cause cell damage. It's been linked to origins of numerous illnesses, including GDM (2012) [87]. It has been proven that GDM women overproduce free radicals and have faulty free radical scavenging mechanisms, both of which are associated with oxidative stress in a hyperglycaemic environment [88]. GLUT4 and IRS-1 interference by ROS prevents insulin-stimulated glucose uptake [89]. In the liver and muscle, ROS also inhibit the synthesis of glycogen. By boosting expression and activity of ROS precursors such as NADPH oxidase 4(NOX4), pro-inflammatory cytokines like TNF- may additionally help oxidative stress. Interestingly, GDM is linked to women who take iron supplements while having a high iron intake [90]. Furthermore, it is thought that homocysteine, a non-protein amino acid produced when methionine is demethylated, is another way that oxidative stress causes GDM. β-cell dysfunction and reduced insulin secretion are caused by homocysteine exposure, even at very low concentrations [91]. A meta-analysis of eleven pertinent studies recently examined the relationship between blood homocysteine levels and GDM. The researchers found that homocysteine concentrations in GDM-affected Women were significantly greater than in non-diagnosed individuals [92]. For homocysteine homeostasis, Folic acid, B2, B6, and B12 are necessary B vitamins. This could be one of the causes of the correlation between GDM and shortages and imbalances in certain micronutrients [93]

## 4 ASSOCIATION OF CARDIOVASCULAR EVENTS IN GDM

**Fig. (4):**
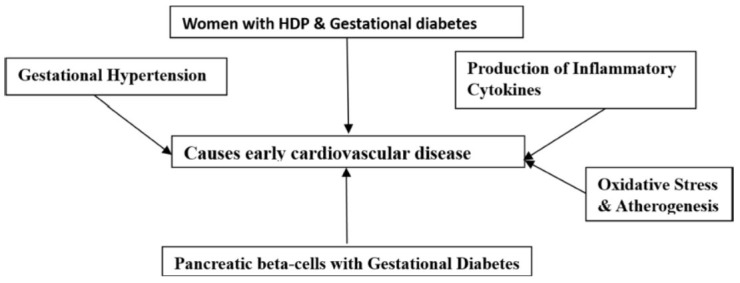
A systematic flow chart of association cardiovascular events in GDM.

A woman's body is under stress throughout pregnancy to adapt to and maintain the fetus's energy needs due to physiological changes. The complex interaction between the child and the mother's surroundings occurs during gestation. The precise etiology of the increased and early CVD risk in women with HDP and gestational diabetes is still unknown. According to the first theory, either a genetic or environmental predisposition increases a woman's risk of cardiometabolic illness before pregnancy. This is corroborated by the discovery that those with gestational diabetes, in particular, have elevated body mass indexes as well as dyslipidemia and other indicators of pre-pregnancy cardiophysiological alterations [15]. The second principle states that the situation of pregnancy itself causes early CVD because of aberrant placentation, inflammation, and endothelial dysfunction [94]. GD increases the risk of developing diabetes with type 2 diabetes because resistance to insulin and declining β-cell function render it hard for the pancreatic β-cells in these women to get over glucose resistance caused by the placenta, which leads to hyperglycemia [68]. Although the link between CVD and pregnancy-related diabetes is not well known, it is possible to speculate that the hyperglycaemic state enhances the production of inflammatory cytokines that encourage oxidative stress and atherogenesis, two factors that contribute to the emergence of CVD [95]. Placental ischemia, a hypoxic state caused by insufficient placental blood flow in women with HDP, is caused by defective placentation [96, 97]. Preeclampsia patients have increased levels of inflammatory cytokines, oxidative stress, and angiogenesis-related modulators, which are thought to cause endothelial dysfunction [97]. Endothelial dysfunction is a pathological condition that affects the entire body and leads to atherosclerosis, which is likely a factor in early coronary artery disease and cardiovascular events [98, 99]. A further effect of gestational hypertension is arterial stiffness, which contributes to the early development of atherosclerosis and CVD (Fig. **4**) [96].

### 4.1 GLUT1-AMPK/ACC Axis in Gestational Diabetes and Fetal Growth Restriction

Macrosomia and fetal growth restriction (FGR) are two fetal problems linked to gestational diabetes mellitus (GDM). Children with GDM-associated FGR are more likely to develop adult-onset obesity and related metabolic diseases. The fundamental processes of FGR linked to GDM have not yet been investigated, though. In this work, ferroptosis and GLUT1 overexpression were seen in the placentas of GDM patients with FGR. In cell models, trophoblast cells were made more susceptible to ferroptosis and GLUT1 expression was triggered by elevated glucose levels. Remarkably, ferroptosis in trophoblast cells under high glucose conditions was markedly inhibited by GLUT1 inhibition. By mechanistically inhibiting AMPK phosphorylation and reducing ACC phosphorylation, increased GLUT1 promoted lipid production and made ferroptosis easier. Treatment with the ferroptosis inhibitor Lip-1 or the GLUT1 inhibitor WZB117 reduced the FGR phenotype in pregnant mice that developed FGR as a result of STZ-induced hyperglycemia. Furthermore, GLUT1 upregulation *in vivo* raised ferroptosis indicators, reduced AMPK/ACC phosphorylation, and produced [100].

### 4.2 Immune Changes in Pregnancy

Changes in the balance of the mother's and fetus's immune systems cause pregnancy issues including gestational diabetes mellitus, hypertension, premature birth, and low birth weight. Timely actions depend on the early identification of biomarkers. Preeclampsia is associated with IL-6, MIP-1β, and IL-12p70, whereas GDM is associated with biomarkers such as IL-1RA, IL-17D, and eotaxin-3. The variables that affect inflammatory indicators include maternal age, BMI, smoking, chronic illnesses, and ethnicity. Birth weight is connected to VEGF and PlGF, but CRP and IL-17 family proteins are linked to severe postpartum hemorrhage. Moderate accuracy was attained by predictive modeling for preeclampsia (AUC 0.672) and GDM (AUC 0.708) [101].

Pregnancy-related preeclampsia raises the chance of developing cardiovascular disorders later on, highlighting the need to evaluate endothelial function, metabolic profiles, and family history to avoid these conditions early. Furthermore, a high first-trimester FT4-to-TSH ratio is linked to an increased risk of GDM, especially in pregnancies with male babies, indicating that early thyroid function monitoring may be useful in predicting and successfully managing GDM risk. In addition to a 2-fold higher risk of coronary heart disease, stroke, and death from coronary heart disease or cardiovascular disease, preeclampsia is linked to a 4-fold rise in future incident heart failure. Our research emphasizes how crucial it is to evaluate continuously cardiovascular risk variables in women who have experienced preeclampsia [52].

## 5 SCREENING FOR GDM

The understanding and diagnostic criteria for GDM are continually evolving and differ across various sources. This diversity stems from numerous factors, including improvements in medical technology, changes in healthcare accessibility, shifts in disease patterns, and regional cultural influences. Consequently, it is generally advised that all expectant mothers undergo a medical evaluation between the 24^th^ and 28^th^ weeks of gestation, or sooner if they present a low-risk profile, to assess their likelihood of developing GDM. If elevated serum glucose levels are detected, subsequent tests such as a 50-gram oral glucose challenge test (GCT) and an oral glucose tolerance test (OGTT) are recommended. The GCT, with a glucose threshold of 140 mg/dl (7.8 mmol/l), produces a positive outcome in 14–18% and 20–25% of women, respectively, with an 80% and 90% sensitivity rate for GDM diagnosis. However, this lower threshold also decreases the specificity by 25% [102]. The American College of Obstetricians and Gynecologists has discovered that the benefits of testing are not uniform across all expectant mothers, even though universal glucose challenge screening is the most effective way to identify GDM. Women are classified as low risk if they satisfy all of following conditions: they are younger than 25 years old, do not belong to an ethnic group with a high incidence of GDM (such as Hispanic, Black, Native Americans, South-east Asian, Pacific Islander, or indigenous Australian), have a Body Mass Index (BMI) under 25, have no prior instances of abnormal glucose tolerance, have never experienced adverse pregnancy outcomes frequently associated with GDM, and have no immediate relatives with diabetes. The Working Group on Diabetes and Pregnancy of the European Association of Perinatal Medicine suggests considering all pregnant women at moderate or elevated risk of GDM, as very few meet the criteria for low risk. Their latest Update and Guidelines on Diabetes and Pregnancy emphasize the importance of OGTT in diagnosing GDM despite the lack of consensus on testing methodology and interpretation. Diagnosis typically requires two or more abnormal glucose readings. The National Diabetes Data Group, established in 1979, recommended a 3-hour, 100-gram OGTT with specific criteria to assess maternal risk. The American Diabetes Association (ADA) now recommends both a 3-hour 100-gram Oral Glucose Tolerance Test (OGTT) and a 2-hour 75-gram OGTT for diagnosing GDM (GDM). These tests share identical diagnostic cut-off criteria for fasting (5.3 mmol/L), one hour (10 mmol/L), and two hours (7.3 mmol/L). However, while the 3-hour test requires two out of four abnormal values for diagnosis, the 2-hour test necessitates two out of three abnormal values. This addition to the guidelines provides healthcare professionals with more options for diagnosing GDM, potentially improving detection and management of the condition during pregnancy [103]. A recent panel by the World Health Organization (WHO) has categorized GDM (fasting glucose a= 7.0 mmol/L or 2-hour glucose = 7.8 mmol/L) as a combined category for diabetes and Impaired Glucose Tolerance (IGT) [104]. Nord *et al.* [105] utilized a 2-hour blood glucose measurement of ≤ 9.0 mmol/L in 75-g OGTT to evaluate the accuracy of diagnosing GDM. They found that there were no significant adverse effects on maternal and newborn outcomes within the borderline range of 8.0-8.9 mmol/L when using the 75-g 2-hour OGTT with a blood glucose limit of ≤ 9.0mmol/L for GDM diagnosis during pregnancy, as opposed to a≤ 8.0 mmol/L. The ongoing Hyperglycemia and Adverse Pregnancy Outcome (HAPO) project, involving 16 centres and around 25,000 pregnant women, aims to address unresolved issues regarding the relationship between varying degrees of glucose intolerance in the third trimester of pregnancy and the risk of adverse outcomes. Stakeholders, particularly those concerned with GDM, anticipate HAPO trial results, hoping for insights into specific causes of complications associated with GDM [106].

## 6 CASE STUDIES

A 6-month cohort study in Melbourne assessed the effectiveness of a multidisciplinary Women's Heart Clinic for women aged 30 to 55 with a history of hypertensive disorders of pregnancy, gestational diabetes, or small-for-gestational-age babies. Among 156 participants, blood pressure control significantly improved (69.2% to 80.5%), and there were notable reductions in systolic blood pressure, body mass index, LDL cholesterol, and total cholesterol. Heart-healthy lifestyle choices also improved. These findings indicate that the clinic significantly enhanced cardiovascular risk factors and potentially improved long-term outcomes for these women [107].

Another systematic review and meta-analysis of 15 studies found that women with a history of GDM had increased risks of cardiovascular and cerebrovascular diseases. Among 513,324 women with GDM, there was a 45% higher risk of overall cardiovascular and cerebrovascular diseases compared to women without GDM. Specific risks included a 72% increase for cardiovascular diseases and a 40% increase for cerebrovascular diseases. Elevated risks were also noted for coronary artery disease, myocardial infarction, heart failure, angina, stroke, and venous thromboembolism. These risks remained significant even when excluding women who developed subsequent diabetes. The evidence quality was rated as low or very low, indicating that GDM is an independent risk factor for these conditions [18].

In this study, Mao *et al.* examined the link between GDM and specific cardiovascular diseases using data from 12,025 women in the National Health and Nutrition Examination Survey (2007–2018). Women with GDM had higher odds of coronary heart disease (CHD) (1.82), heart failure (1.43), and stroke (1.19) compared to those without GDM. T2D significantly contributed to these risks, explaining 43.90% of CHD, 67.44% of heart failure, and 63.16% of stroke excess odds. Combined with previous studies, the odds ratios were 1.81 for CHD, 1.66 for heart failure, and 1.25 for cerebrovascular disease. The study found that GDM was more strongly associated with CHD and heart failure than cerebrovascular disease, partly due to T2D [108].

Tcheugui *et al.* used Ontario's healthcare databases to examine the link between gestational hypertensive disorders (GHTD), gestational diabetes (GD), and cardiovascular disease (CVD) in 886,295 women with live births between 2007 and 2018. Women with pre-existing diabetes, hypertension, or CVD were excluded. Over 12 years, 1,999 CVD events occurred. In the first 5 years postpartum, isolated GHTD was associated with higher CVD risk (aHR 1.90), while GD alone or combined with GHTD was not. After 5 years, both isolated GHTD (aHR 1.41) and combined GHTD and GD (aHR 2.43) increased CVD risk, but GD alone did not. The study suggested a need for targeted CVD preventive care for women with both GD and GHTD [109].

A meta-analysis of nine studies involving 5,390,591 women found that those with GDM had a twofold increased risk of future cardiovascular events compared to those without GDM. This risk was independent of developing type 2 diabetes, with GDM alone linked to a 56% higher risk of cardiovascular events when excluding women who later developed T2D. Additionally, women with GDM had a 2.3-fold increased risk of cardiovascular events within the first decade postpartum. The findings highlighted GDM as a significant risk factor for cardiovascular disease, emphasizing the need for early monitoring and intervention [17].

In a follow-up study of women who delivered between 2008 and 2009, those with a history of gestational diabetes mellitus (GDM) showed more adverse cardiovascular and vascular outcomes at an average age of 38. These women had higher rates of prediabetes or diabetes, along with greater impairment in endothelial function and cardiac structure. Specifically, they experienced increased septal wall thickness and worse diastolic function. Some risks persisted even after adjusting for prediabetes or type 2 diabetes, underscoring the need for better preventive measures for cardiovascular health in women with prior GDM [110].

## 7 VARIOUS TREATMENT APPROACHES

Managing cardiovascular disease risk in pregnant women with GDM involves a comprehensive approach that includes regularly monitoring blood glucose levels and blood pressure, lifestyle changes such as a heart-healthy diet and regular exercise, and timely treatment if necessary. Effective glycemic control is crucial to prevent T2D and associated cardiovascular problems. Postpartum women should be screened for T2D and cardiovascular risk factors and educated on maintaining a healthy lifestyle. Medications for blood pressure and cholesterol may be used if lifestyle changes are not enough, and ongoing monitoring of cardiovascular health is important to address the increased long-term risk associated with GDM [111].

## CONCLUSION

Pregnancy's physiological demands, which serve as a stress test for cardiometabolic diseases, might determine whether women are at risk for cardiovascular illnesses and what factors may lead to physiological changes that accelerate the onset of plaque. Diabetes with gestation and HDP placed early and late postpartum women at risk for CVD. However, there is little knowledge of this danger among women and medical professionals. Interventions are continuously being developed and evaluated, with the main goals being to enhance screening, encourage lifestyle changes, and discover methods to identify CVD with early onset. However, much of this research is still in its early phases, and it will take a long time to incorporate it into practical guidelines for clinical practice. All that's certain is that for doctors to identify the increased risk of CVD associated with HDP and GD, they must receive enough education. CD and risk factor screening must start after an impacted pregnancy and should be continued for the duration of a woman's life.

## Figures and Tables

**Fig. (1):**
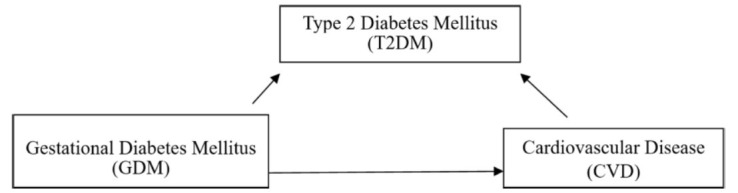
A simple relationship chart showing the possible connections between cardiovascular illness, glucose type 2, and GDM.

**Fig. (3):**
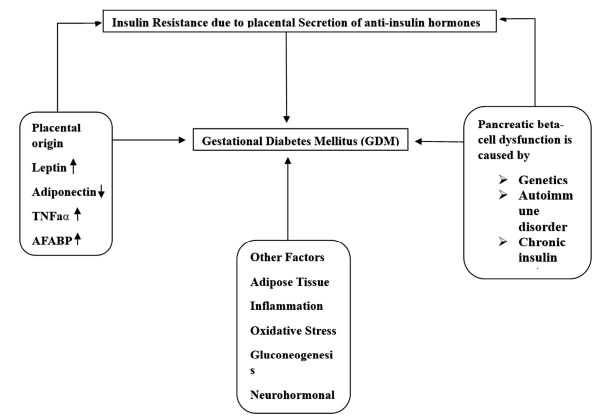
A systematic flow chart of the pathogenesis of gestational diabetes and their relative factors.

## LIST OF ABBREVIATIONS

BMI: Body Mass Index
CV: Cardiovascular
CVD: Cardiovascular Disease
GD: Gestational Diabetes
GDM: Gestational Diabetes Mellitus
MS: Metabolic Syndrome
T2D: Type 2 Diabetes
T2DM: Type2 Diabetes Mellitus
